# Exploring Factors Contributing to the Smoking Behaviour among Hong Kong Chinese Young Smokers during COVID-19 Pandemic: A Qualitative Study

**DOI:** 10.3390/ijerph19074145

**Published:** 2022-03-31

**Authors:** Katherine-Ka-Wai Lam, Ka-Yan Ho, Cynthia-Sau-Ting Wu, Man-Nok Tong, Lai-Ngo Tang, Yim-Wah Mak

**Affiliations:** School of Nursing, The Hong Kong Polytechnic University, Hong Kong, China; kw-katherine.lam@polyu.edu.hk (K.-K.-W.L.); cynthia.wu@polyu.edu.hk (C.-S.-T.W.); man-nok.tong@polyu.edu.hk (M.-N.T.); lai-ngo.tang@polyu.edu.hk (L.-N.T.); yw.mak@polyu.edu.hk (Y.-W.M.)

**Keywords:** smoking behaviour, young smokers, pandemic, COVD-19, qualitative

## Abstract

COVID-19 has significant impacts on young smokers in their smoking behaviors. This qualitative study summarises the lived experience of young smokers during COVID-19. Moreover, through their lived experience, we aim to understand how the COVID-19 pandemic influence tobacco use behaviours in this population. A purposive sampling of 48 smokers aged between 17–25 years old is individually interviewed for 30 to 45 min. All interviews are transcribed in verbatim and analysed by two researchers separately using Colaizzi’s method of descriptive phenomenology. The results reveal the following six important themes, which could explain the mixed pattern of smoking behaviour changes in young smokers: (1) perceptions of COVID-19 and its association with smoking, (2) more time at home, (3) taking masks off to smoke, (4) the effects of COVID-19 on smokers’ financial status and academic performance, (5) reduced social gatherings, and (6) restricted access to tobacco products. To conclude, this pandemic and the anti-pandemic measures, i.e., mask mandates, stay-at-home and work-from-home orders, and class suspension, result in both new obstacles and new advantages for smoking cessation among young people. More studies should be performed to monitor any transition of tobacco products and the trajectory of use in this population during this pandemic, thus informing public health policy making.

## 1. Introduction

The novel coronavirus disease (COVID-19), an infectious respiratory disease caused by the SARS-CoV-2 coronavirus, has become a worldwide pandemic since December 2019 [1], resulting in overwhelming and profound consequences for human life [1]. COVID-19 has led to 256 million reported cases and 5.1 million deaths worldwide as of 23 November 2021 [2].

Smoking might be a risk factor for COVID-19 infection [3]. According to the World Health Organization [3] and recent evidence [4,5], smokers are more likely to contract COVID-19 because smoking involves repeated interactions between the hands and the mouth, leading to an increased chance of contamination. In addition, although COVID-19 is transmitted through droplets and physical contact [6], burning tobacco can generate aerosol particles that harbour and transmit COVID-19 under some specific circumstances [7]. Additionally, as a result of the harmful effects of smoking on the respiratory and immune systems, smokers are prone to developing severe complications and have a more rapid disease progression after contracting COVID-19 [8].

To control the spread of COVID-19, governments worldwide have introduced numerous and varied anti-pandemic measures. Examples include issuing mask mandates in public; encouraging frequent hand washing; encouraging residents to stay and work at home restricting the number of people in gatherings; closing shopping malls, restaurants, and bars; requiring quarantine before or after travel [9]. However, some of these measures significantly affect smoking behaviours. For example, concerning mask mandates, smoking is not a reasonable excuse to remove a mask in public [10], creating a barrier to smoking. Moreover, since mask mandates are considered a key measure to prevent the spread of COVID-19, smokers who remove their mask to smoke in public may be socially stigmatised [11]. In addition, the closure of some public places, e.g., bars and restaurants, reduces the available venues for smoking, thus displacing smokers to smoke in other places [12].

The psychological distress resulting from the COVID-19 pandemic may also affect smoking behaviours. Because of the high mortality rates and uncertain prognosis, the general public, including smokers, are anxious about themselves, their families and friends contracting COVID-19 [13]. Previous studies show that such anxiety may exert a double-edged impact on smokers [14]. A nationwide survey of 4075 smokers in the United Kingdom observed that the deterioration of mental health and psychological well-being was associated with increased smoking because smoking was used as a coping mechanism by smokers during the pandemic [15], whereas Bommelé et al. [14] observed that the threat of contracting COVID-19 and its associated complications motivated smokers to improve their health by reducing or even quitting smoking.

An increasing number of studies have confirmed the aforementioned postulations and indicated a mixed pattern of smoking behavioural changes in response to COVID-19 [14,16]. In a longitudinal survey of 6014 smokers, 14% quit smoking during the pandemic. Of those who continued smoking, 68% reduced, 14% maintained, and 18% increased cigarette consumption [16]. Another survey of 957 smokers similarly found that 14.1% smoked less, 18.9% smoked more, and the remaining maintained the same cigarette consumption during the pandemic [14].

Notwithstanding the increasing number of quantitative studies showing that the COVID-19 pandemic has had a significant impact on smokers, scant qualitative evidence has summarised their lived experience during COVID-19 or contributed to understanding how their lived experience influences their tobacco use. A literature review found several qualitative studies that explored the attitudes and motivations toward quitting among smokers in this pandemic. However, these studies were conducted on older people [17] and hospitalised patients [18], not younger smokers who should warrant special attention as they are likely to continue their smoking habit into adulthood [19]. To address the gap in the existing literature, this qualitative study aimed to summarise the lived experience of young smokers during COVID-19. Moreover, through their lived experience, we aimed to understand how the COVID-19 pandemic influenced tobacco use behaviours in this population.

## 2. Materials and Methods

### 2.1. Design

This study used a descriptive phenomenological approach to provide a thick description of the lived experience of smoking under COVID-19 among the young [20]. Thick description refers to a detailed description of the process, context, and people in the research, including the meaning and intentions of respondents and researchers’ conceptual developments so as to provide a basis for readers to evaluate the data quality [20]. Descriptive phenomenological approach was used to describe the lived experience of smokers under COVID-19. This method enables researchers to obtain the meanings from the rich descriptions, which can be related to the phenomenon of people in comparable situations [21]. This is particularly useful for studying the smoking behaviours during COVID-19 in young smokers with rich descriptions.

### 2.2. Setting

This was an extension of a cross-sectional study that had been previously conducted [22]. The cross-sectional study aimed to examine the patterns of change in smoking behaviours before and during COVID-19 among 348 young smokers aged 12–25 years recruited from smoking hotspots in Hong Kong urban areas. Smoking hotspots are places where smokers gather to smoke. This qualitative study was conducted during January and March 2021, which was around a year after the epidemic outbreak. During most of the time in the study period, Hong Kong residents were asked to follow strict measures by the government such as wearing masks mandatorily in public areas, restricting the number of people in each table of the restaurants to 4, early closure of the restaurants at 6 pm daily, stopping the flights from high-risk areas landing in Hong Kong, and compulsory quarantine after arriving from other countries. At the end of March 2021, the total number of confirmed cases in Hong Kong was 11,461. More than 490,000 doses of vaccine have been administered in Hong Kong after the launch of the vaccination programme in mid-February 2021. A structured questionnaire was administered by a research assistant to collect with their demographic characteristics and smoking behaviours.

### 2.3. Respondents

Respondents who participated in the previous cross-sectional study were invited to participate in this study. To identify suitable respondents for this study, the technique of maximum variation, which is a type of purposive sampling, was used. In the previous cross-sectional study, all participants were asked about their smoking consumption before and during COVID-19. Participants were then categorised by any change in their smoking behaviour into the following three groups: increased, decreased, or unchanged smoking consumption [22]. In this qualitative study, we purposively selected and invited smokers from each of the three categories. Through using the technique of maximum variation, researchers could ensure that the recruited smokers for interviews would have very different on smoking behaviour, increasing the diversity of the interviewees. This can allow researchers to capture the phenomenon, i.e., the COVID-19 pandemic influenced tobacco use behaviours in this young smoker population during this study period, by observing and identifying significant statements or patterns that merely existed in smokers who increased smoking consumption, but not in those who decreased or unchanged their consumption, or vice versa. As a result, researchers were able to ascertain that the statements or patterns identified truly described and were related to the central phenomenon, i.e., tobacco use behaviours affected by the COVID-19 pandemic. The sample size was determined by data saturation, which occurred after interviewing 48 respondents, with 8, 19, and 21 having the same, increased, and decreased cigarette consumption, respectively, during COVID-19.

### 2.4. Data Collection

Based on the previous cross-sectional study [22], respondents’ demographic data and important smoking-related data such as the results of Fagerström test and stages of readiness to quit were extracted. All respondents were separately interviewed by two research assistants. One was an interviewer, and another was an observer. Each interview was audio-recorded and lasted approximately 30 to 45 min in a meeting room at a local university. A semi-structured interview guide was developed by our research team to guide the interviews. The following four areas were included in the guide: (1) smoking profiles; (2) perception of COVID-19; (3) any change in smoking behaviour during COVID-19 and possible reasons; (4) impacts of COVID-19 on their daily activities. To elicit more qualitative data, probing was used. Before the commencement of the interviews, the two research assistants were trained by our research team on communicating with young smokers. Importantly, they were asked to disregard their preconceptions throughout the interviews.

### 2.5. Data Analysis

All interviews were transcribed in verbatim and analysed by two researchers (Katherine-Ka-Wai Lam and Ka-Yan Ho) using Colaizzi’s method of descriptive phenomenology, which consisted of distinctive steps to provide a rigorous analysis to produce a concise but thick description of the phenomenon for the study [23]. The two researchers analysed all the interviews separately. First, the researchers repeatedly read the transcripts and listened to the audiotapes to immerse themselves and obtain a general sense of lived experience of the respondents. Relevant statements to the research questions were extracted from the transcripts. The researchers then formulated the meanings of the relevant statements to better express the feelings of the respondents. The researchers regularly compared the formulated meanings with the extracted statements to search for any discrepancy. Then, categories, clusters, and themes were identified and organised into comprehensive descriptions. To ensure that the descriptions could truly reflect respondents’ lived experience, respondents were asked to provide comments on the descriptions. Research team meetings were conducted to resolve any discrepancy throughout the analysis.

### 2.6. Research Rigour

Several strategies were employed to promote the rigour of the interviews [24]. To ensure transferability, rich quotes and descriptions relating to the respondents’ lived experience of smoking behaviour under COVID-19 were provided when reporting the results. Dependability and credibility were promoted by asking the two same and well-trained research assistants to conduct all interviews and training the research assistants to communicate with smokers by our research team. All of our team members had rich experience in conducting qualitative studies on smokers. In addition, the technique of triangulation was adopted [25] by asking one research assistant to be an interviewer and another as an observer to check for any nonverbal cues such as respondents’ facial expressions, gestures, and postures to increase the richness of qualitative data and gather valuable insight from the respondents. Regular meetings were organised among the research team members and research assistants to discuss any subtleties from respondents by checking the observational data. This could enable the team to obtain a comprehensive understanding of the data and avoid any misinterpretations [26]. Moreover, the research assistants were reminded to be open-minded. Particularly, instead of replying to the respondents that the responses were normal and rejected the data as subjective feelings, respondents were always encouraged to express themselves freely throughout the interviews. To provide privacy and avoid interruptions, all interviews were conducted in a quiet and safe room at a local university. Besides, to further ensure the accuracy of the data, field notes were written during the interviews by the interviewers. The field notes contained details of time, date, settings, observations, and key conversations of the interviews. The contents of the interviews were audio-recorded to minimise recall bias and errors. To verify that the codes and themes could truly reflect respondents’ experiences, any new data was regularly compared to all data and checked with respondents. Apart from transferability, dependability, and credibility, ensuring confirmability is also required in promoting rigour. Since any pre-existing beliefs and perceptions may lead to bias-ridden judgements and consequently affect the accuracy of the findings [24], two researchers (Katherine-Ka-Wai Lam and Ka-Yan Ho) who analysed the data were asked to reduce such bias by recalling and making pre-existing beliefs and perceptions explicit and applying bracketing throughout the analysis to disregard their preunderstanding from any similar research. Member checking was also conducted. The analysed data was returned to participants to check for accuracy. Any inconsistencies or ambiguities were resolved in regular research meetings after the analysis.

### 2.7. Ethical Considerations

Ethical approval (HSEARS20201118001) was obtained from the institutional review board of the Hong Kong Polytechnic University. Our research team strictly adhered to the principles laid down in the Declaration of Helsinki (https://www.wma.net/policies-post/wma-declaration-of-helsinki-ethical-principles-for-medical-research-involving-human-subjects/) (accessed on 1 December 2021) throughout the study. Importantly, before obtaining informed consent, respondents were fully notified about the study’s purpose and procedures. They were told that they could choose freely to participate or not to participate in this study. They could withdraw from the study at any time without penalty. To protect confidentiality, parental consent was not sought even when the respondents were under the age of 18. This is because these respondents might not disclose their smoking habits to their parents. Seeking parental consent would then disclose this sensitive issue to their parents, beaching the confidentiality. Moreover, all data was stored in a locked place that could only be accessed by our research team.

## 3. Results

### 3.1 Demographic Characteristics of Respondents

Table 1 shows the demographics of the respondents, of whom 70.8% (34/48) were male, 56.3% (27/48) studied in subdegree, 56.3% (27/48) were full-time students, and 93.8% (45/48) were single. The mean age was 21.4 years (SD: 2.8). Concerning their smoking profile, the mean age at smoking initiation was 15.8 years (SD: 2.9) and the mean smoking duration was 5.7 years (SD: 3.4). In total, 56.3% (27/48) reported smoking traditional cigarettes in the past 30 days during pandemic, 35.4% (17/48) smoking electronic cigarettes and/or heated tobacco products, and 22.9% (11/48) were dual users. For those who smoked traditional cigarettes, their mean daily cigarette consumption was 8.22 (SD: 6.23). According to the Fagerström test, 58.3% (28/48), 27.1% (13/48), and 14.6% (7/48) had mild, moderate, and severe nicotine dependence, respectively. In terms of stages of readiness to quit, 58.3% (28/48) were in pre-contemplation, followed by 27.1% (13/48) in contemplation and 14.6% (7/48) in preparation. Of the 48 respondents, 8 (16.7%) maintained their cigarette consumption during COVID-19, and 39.6% (19/48) increased, and 43.8% (21/48) reduced smoking. Moreover, 60.4% (29/48) of respondents had made previous quit attempts.

#### Factors Contributing to Smoking Behaviours

The themes, subthemes, and examples of quotes from the respondents in the interviews were presented in Supplementary Materials (see Table S1). 

Theme 1. Perceptions of COVID-19 and its association with smoking

Subtheme 1. Relationship between COVID-19 and smoking

Most of the respondents agreed that COVID-19 is a serious disease. However, they said that the mortality cases were mainly from older people and those with chronic diseases. Given that they were relatively young, their risk of death was low. Some respondents also thought that they could fully recover from COVID-19 even if they were to be infected. Some said the outbreak in Hong Kong was well-controlled when compared with other countries, with only a few reported cases each day. Hence, the chance of infection was low. Because the respondents perceived themselves as not being vulnerable to COVID-19, they said they did not plan to reduce or quit smoking, even if smoking could increase their chance of infection. As one respondent (Respondent M3, male, aged 20) stated in the semi-structured interview, “COVID-19 is serious, but not many young people die because of that. Only older people and those with chronic diseases may die after infection. I think the chance (for me) to get COVID is small… (Have you ever heard that smoking can increase the chance of COVID-19 infection?) Yes. Someone said so. (What do you think?) I don’t have many feelings about this. (Can you tell me a bit more?) As I mentioned, not many young people will die after being infected with COVID-19. People said smoking could increase the risk of getting COVID. How big could the risk be? That’s why I don’t bother.”

Moreover, a majority of the respondents said they had never heard that smoking could increase the risk of COVID-19 infection. Even when some mentioned that they knew about this issue, they thought the risk was small when compared with other activities, such as gathering with friends and dining in restaurants. Hence, they thought such a minor risk could be disregarded and continued to smoke. As described by one respondent (Respondent M5, female, aged 22), “Yes, the government said smoking can increase the chance of getting COVID. However, there are many things which can increase the risk of infection. For example, going out with friends, or waiting for a bus at the bus stop. You can only avoid every risky activity by staying at home. This is impossible. That’s why I continue to smoke.”

Subtheme 2. Protect family members from infection

Some respondents said they did not worry about being infected with COVID-19 themselves because they were young. However, they were concerned for some of their family members, e.g., parents or children with chronic diseases, making them susceptible to COVID-19 infection and its severe complications. These respondents said they knew smokers had a higher chance of being infected with COVID-19. Hence, they were worried that they might spread the infection to their loved ones if they were to be infected. They therefore decided to reduce or even stop smoking during this pandemic to protect their family members. As indicated by a respondent (Respondent D12, male, aged 23), “I personally don’t worry about infection. Even if I am infected, the possibility of death is low as I am young. (But why did you decide to quit smoking?) My father has multiple chronic diseases—diabetes and hypertension. I heard from the news that smokers are more likely to be infected with COVID-19. I don’t want to be infected and then pass the disease to my family, especially my father. That’s why I stopped smoking.”

Theme 2. More time at home

Subtheme 1. Easier to access tobacco-related products

Many respondents said they had spent more time at home during this pandemic due to the following: (1) worry about infection; (2) work-from-home policies; (3) class suspension; (4) no in-restaurant dining after 6:00 pm; (5) earlier closure of shops. As such, they had easier access to tobacco-related products, e.g., cigarettes, lighters, and ashtrays, and increased smoking. In the semi-structured interview, Respondent I6 (female, aged 19) said, “I smoked more in this pandemic… Because I stay at home most of the time. […] Because of class suspension, I don’t have to go to school. Previously, even if I wanted to smoke, I couldn’t do that because it breaks the school rules. However, when I am at home, I can smoke whenever I want. I can easily reach cigarettes. Hence, I smoke more.”

Subtheme 2. Boredom

Some respondents said they increased smoking because they were bored when staying at home. They told us that they performed some activities, e.g., playing mobile games and watching YouTube videos, to kill time. When doing these activities, they unconsciously smoked and hence increased smoking. Respondent I12 (male, aged 23) provided the following description: “Because classes have been suspended, I have spent most of the time at home. However, staying at home is boring. Sometimes, I watch YouTube videos to kill time. When watching the videos, I always smoke. […] No particular reason; I just smoke unconsciously when watching videos. Habit. Hence, I smoked more.”

Subtheme 3. Increased family conflicts

Many of our respondents reported that family conflict was an important factor that increased tobacco use during COVID-19. In particular, the respondents said that the government encouraged people to stay and work from home rather than go outdoors. In addition, some respondents mentioned that some family members were laid off during the pandemic. As a result, their family members had more time to stay at home during COVID-19. This resulted in more arguments and conflicts, at times leading to an unhappy, intense, and stressed environment in the family. As such, the respondents said they used smoking as a coping mechanism to manage the stress associated with these family conflicts. As described by Respondent I8 (male, aged 20), “My relationship with my father is not good. However, the situation was still acceptable as previously he had to go out for work, and we didn’t meet each other frequently. […Now it is] Worse. Due to COVID, he stays at home most of the time. I also stay at home because my school has been closed. So my father always picks on me regardless what I do. I feel so stressed and annoyed, so I smoke more.”

Subtheme 4. Reduced smoking because family members do not know I smoke

Some respondents said they had reduced their smoking as they had spent more time at home in this pandemic. Because their families did not know about their smoking habit, they could not smoke at home, which in turn reduced their smoking. As indicated by Respondent D4 (female, aged 18), “My parents do not know I am a smoker. As I am required to attend online classes via Zoom at home, I cannot go outside to smoke, and hence reduced smoking.”

Theme 3. Taking masks off to smoke

Subtheme 1. This is illegal

A majority of respondents reduced smoking due to mask mandates. Specifically, removing masks in public areas for smoking was made illegal and could result in a warning, a fine, or a summons from the police; hence, these respondents said they dared not do so. As described by Respondent D9 (male, aged 21), “I have reduced smoking in this pandemic. […] I used to smoke on the street because my parents did not know of my smoking habit. However, that has become difficult since the pandemic. As you know, taking off masks is illegal. The police patrols frequently on the weekends. If they find you taking off masks for smoking, you have to pay $5000 [as a fine]. This is a lot for me. Hence I have reduced smoking.”

Subtheme 2. Inconvenience

Some respondents mentioned that they reduced smoking because of the inconvenience caused by mask mandates. Particularly, they had to smoke using one hand while the other hand held the removed mask. Some also mentioned that it was inconvenient to prevent the mask from being contaminated during smoking. As described by Respondent D15 (female, aged 24), “I now seldom smoke on the street. […] Very inconvenient. […] Now, all people in Hong Kong are required to wear masks [in public areas]. If I smoke, I have to take off my mask, and hold the mask throughout smoking. On one occasion, I dropped my mask on the street when I smoked. And I forgot to bring another mask with me. Luckily, a smoker gave a new one (mask) to me. It is so inconvenient. Since then, I haven’t smoked in the street. I am not someone who must smoke.”

Subtheme 3. Social stigma

A few respondents mentioned that they reduced smoking because of the social stigma. They said that wearing a mask became the norm in Hong Kong; those who did not wear or take off masks on the street were stigmatised by the public. The ways of stigmatisation included (1) staying far away, (2) fast walking, (3) covering their mask with their hands, (4) reminding companion(s) that there was someone not wearing the mask, and (5) gossiping with companion(s). As indicated by Respondent D18 (male, aged 25), “Almost 100% of people in Hong Kong wear a mask. People will stare you if you do not wear mask regardless of any reason. If you take off your mask on the street, the surrounding people will stay far away and walk very fast. This feeling is not good.”

Theme 4. The effects of COVID-19 on smokers’ financial status and academic performance

Subtheme 1. Quit or reduced smoking to save money

Some respondents said they reduced smoking because of financial issues related to the pandemic. They mentioned that they lost their jobs due to the economic downturn, or if they still had a job, they were forced to take no paid leaves. As such, their incomes had been reduced. Other respondents said they would like to save money because they were uncertain about their jobs. Even though they were still employed, they might be made redundant without warning. Based on these issues, the respondents said they tried to cut down on other expenses, such as smoking. Respondent D21 (male, aged 25) provided the following descriptions: “My job is not secure. […] I am working in the retail industry. My company has been greatly affected after the border was closed. Some of my colleagues were made redundant. I don’t know how long my company can survive. So, I want to save more money in case I am made redundant. Cigarettes are expensive. The only way is not to buy so I can save money. I won’t die if I don’t smoke.”

Subtheme 2. Smoking offers respite from financial distress

Some respondents who experienced financial hardship reported increased smoking. They mentioned that they encountered this hardship for various reasons, including layoffs, reduced income, and unsecured jobs. Some respondents who were new graduates also told us that they were frustrated by the difficulties of finding a job in this pandemic. They felt stressed and distanced themselves from associates because of their low self-esteem. As indicated by Respondent I3 (male, aged 18), “I think I am useless. I have kept sending out a lot of applications but have received few responses. I don’t want to talk to other people, especially my parents, because I don’t want them to worry about me. When I think about this issue, I feel breathless. Life is too hard for this [millennial] generation.” Under these circumstances, some respondents said they smoked more because smoking was the only way to cope with stress. A Respondent (I14, female, aged 23) said, “There’s nothing you can do. All people in the world face the same situation. [Did you talk to your friends?] Meaningless. Things would not be improved. [So you smoked?] Yes. This is the only thing that I can do. It provides me a space to breathe. I can stop thinking about this issue (redundancy) for a while. [So this makes you smoke more?] Yes.” These respondents also said they switched to purchasing counterfeit cigarettes to save money.

Subtheme 3. Worry about their performance in public examinations

A few respondents said they were worried about their performance in public examinations because they were unable to have normal instruction due to the suspension of classes. Some also felt anxious because they did not know when face-to-face teaching could resume. Given these issues, they felt stressed and smoked more than usual. As described by Respondent I1 (male, aged 17), “I hope everything can return to normal as soon as possible because I have to attend the DSE [Hong Kong Diploma of Secondary Education, a public exam] next year. But now, we can only go to school for half-days. This is not enough for us to prepare for the DSE. I am so worried that I cannot do well in the DSE and then will not get an offer from the university. This makes me stressed, and I smoke more.”

Theme 5. Social gatherings

Subtheme 1. Reduced smoking due to less peer pressure

Most of the respondents who reported reduced smoking consumption mentioned that their smoking peers used to always ask them to smoke during social gatherings. They felt it was hard to reject these requests because of peer pressure. They were worried about being ostracised if they did not smoke. Thus, they used to smoke every time when hanging out with their smoking peers. After COVID-19, however, the respondents said they were less likely to meet their smoking peers, especially due to the suspension of classes. This in turn reduced peer pressure, consequently reducing their smoking consumption during COVID-19. As described by Respondent D6 (male, aged 19), “Before COVID, I was always smoking with my peers because they asked me to in the gatherings. They asked me to smoke, and so I smoked. […] I thought of [not smoking] before. But I was so scared that they would not invite me to play the next time. I do not want to be alone. [Why would you reduce your smoking consumption during COVID-19?] Actually, I did not think of reducing smoking. However, during COVID, my parents asked me not to go out so often. Also, the school has been closed. So I have fewer gatherings with them [smoking peers]. That’s why I smoked less.”

Subtheme 2. Increased smoking due to less social support

On the contrary, some respondents reported that they increased their smoking consumption during the pandemic because they received less social support. They said they had friends who supported quitting; whenever they met these friends, the friends would advise them not to smoke and remind them of the health consequences of smoking. If they smoked, their friends would stay away from them or even leave the gatherings. This social influence motivated the respondents to reduce smoking or even quit smoking. Respondent I10 (female, aged 21) said, “I used to smoke less when meeting friends because they always asked me not to smoke in front of them. Also, they kept saying that smoking was not good for my health. They were annoying, but I knew that they just did it for my sake. So I listened (to them) and smoked less.” However, during this pandemic, the respondents said they associated less with these friends, and hence smoked more due to fewer reminders. One respondent (I13, female, aged 23) told us, “After COVID, I met them less often. Maybe one time a month. I did not intend to smoke more. But there was no one to remind me [not to smoke], hence I smoked whenever I wanted. So it seems that I smoke more now.”

Theme 6. Access to tobacco products

Most respondents who smoked electronic cigarettes (EC) and/or heated tobacco products reported decreased smoking or switching to conventional cigarettes during this pandemic. As reported by the respondents, the major reason is that in Hong Kong EC and heated tobacco products were less popular than conventional cigarettes, which are available in convenience stores. EC and heated tobacco products were normally available in checkered shops only. However, due to the pandemic, most shopping malls closed earlier, hence EC and/or heated tobacco products were inconvenient to purchase. They therefore decided to smoke less or switch to conventional cigarettes for reasons of convenience. As described by Respondent I14 (female, aged 23), “It is difficult to buy EC in the pandemic. They are only available in checkered shops in MongKok. However, the shops now close at 8:00 pm due to less foot traffic. Because I get off work at 6:00 pm, it would be a rush for me to buy EC. So I switched to smoking conventional cigarettes.”

Table S1 shows an abridged table of themes, subthemes, and examples of quotes from the interviews.

## 4. Discussion

In these semi-structured interviews, quite a number of respondents said that their smoking was not associated with COVID-19. In addition, they generally considered themselves to be less vulnerable to COVID-19 compared with older people and those with chronic diseases. Specifically, the respondents mentioned that they believed they could recover quickly and fully from COVID-19 even if infected. These qualitative findings reflect the fact that young smokers perceive a lower vulnerability to COVID-19 infection and its associated health consequences when compared with adult smokers in general. A possible explanation is that young people are less competent in identifying health risks and tend to judge themselves as having a minimal chance of developing negative health outcomes because of their better physical health and the remoteness of the potential outcomes [27]. Rigotti et al. [28] found that perceived vulnerability to COVID-19 is a key factor that influences smokers’ motivation to quit smoking, with those perceiving a higher risk of infection being more motivated to quit. Because young smokers perceive a lower level of vulnerability, it is understandable that a majority of them opted to maintain their daily cigarette consumption during COVID-19, as shown in our previous quantitative study [22].

Our semi-structured interviews also revealed that COVID-19 has greatly affected the economy, and young smokers have encountered financial distress due to various reasons, including layoffs, reduced income, and insecure jobs. Most of our respondents were recent graduates with little working experience who were having difficulty finding jobs in the pandemic. These qualitative findings extend our understanding from a previous cross-sectional survey that observed a significantly higher unemployment rate in people aged 20–25 than among those aged 31–35 [29]. Kalkhoran, Levy and Rigotti [30] found that increased worry about financial problems was associated with increased cigarette consumption. However, our qualitative findings add to that study by showing that financial distress could have the following double-edged effect on tobacco use among young smokers: Some respondents specifically said that smoking was a coping mechanism to help distract them from financial stress, whereas some mentioned that they quit or reduced smoking because they wanted to save money. More studies should therefore be conducted to further clarify the financial impact on smoking during COVID-19.

In addition, this study revealed that mask mandates may result in reduced smoking in public areas. As was evident in our semi-structured interviews, many respondents said they reduced smoking because taking masks off to smoke was illegal under mask mandates; they also felt inconvenienced or stigmatised if they were to take off their masks to smoke. This may be explained by a phenomenon specific to Hong Kong. Due to prior experience with the severe acute respiratory syndrome (SARS) outbreak in 2003, the public was on high alert for a similar occurrence, with 96.6% compliance with face mask use reported during COVID-19 [31]. Hence, universal mask wearing is considered a social norm. This is in contrast with societies that may have difficulty accepting mask wearing due to fear of identifying with non-indigenous culture and religious practise [32]. As such, it is understandable why our respondents said they were reluctant to smoke in public areas because of the stigma attached to taking off the mask. Although stigmatisation seems to have helped reduce smoking in public areas, Helweg-Larsen, Sorgen and Pisinger [33] observed that it does not help smokers abstain from cigarettes, as stigmatisation leads to shame and rejection, which moves them away from quitting, rather than toward it. Also, smokers may conceal their smoking behaviours in public due to this perceived stigma, resulting in the displacement of smoking from public areas to households, in turn increasing second-hand and third-hand smoke exposure among smokers’ families. In fact, a recent study in Israel showed that one-third of adult smokers increased the tobacco exposure of other household members during the pandemic [34].

Apart from mask mandates, other anti-pandemic measures, particularly stay-at-home and working-from-home orders, were found to affect young smokers’ daily lives, exerting double-edged effects on their smoking behaviours. As shown in our semi-structured interviews, some respondents increased their smoking consumption because staying at home was “boring”, with increased time exposed to smoking trigger objects such as cigarettes and lighters. Stay-at-home and work-from-home orders also led to increased family conflicts, and some respondents consequently increased smoking to distract themselves. These qualitative findings provide a potential explanation to address the results of a large-scale population survey that observed an increase in cigarette consumption after the implementation of the lockdown order in California [35]. Despite the aforementioned negative effects, stay-at-home and work-from-home orders could, to some extent, reduce smoking and even lead to cessation in smokers who are unable to smoke in the home. This is particularly true for young smokers who are 18 years old or younger because Hong Kong legislation bans the sale of cigarettes to minors [36]; hence, minors may tend to conceal their smoking habits from their parents due to potential punishment [37]. This is supported by our qualitative findings, in which some respondents reported that after the stay-at-home order was implemented, they reduced their daily cigarette consumption because their families did not know about their smoking habit.

Our semi-structured interview revealed that some respondents switched between different tobacco products due to the inconvenience of purchasing them after the implementation of anti-pandemic measures. Moreover, because of financial distress, some respondents admitted to buying smuggled cigarettes that were much cheaper. These qualitative findings support the findings of a cross-sectional survey of 2167 youths and young adults using electronic cigarettes that reported that 2.3% switched to other forms of nicotine such as combustible cigarettes, nicotine patches or gum, and 4.6% to other products during COVID-19 [38]. Our findings and the results from previous studies reflect that COVID-19 and the related anti-pandemic measures may affect access to tobacco products and the point of sale, resulting in transitions between different tobacco products among young smokers. Hair et al. [39] showed that young smokers frequently switched between different tobacco products, and that the current state of use strongly predicted the future state within a year. More studies should therefore be carried out to map the transition among tobacco products and the trajectory of use in this population during this pandemic, which can further inform the priorities of tobacco control policies [39].

Additionally, some respondents reported reducing their cigarette consumption because they were less likely to spend time with their smoking peers due to the suspension of classes during COVID-19. Our findings are in line with a meta-analysis that reported that peers′ opinions and behaviours play an important role in smoking initiation and continuation among youth and young adults [40]. However, some respondents surprisingly indicated in the semi-structured interviews that they actually increased their cigarette consumption during COVID-19 because they received less social support from non-smoking peers. This qualitative evidence suggests that anti-smoking messages delivered by peers could assist young smokers in reducing or even quitting smoking. Because most studies have mainly focused on the negative impacts on young people’s smoking [40], more studies should be carried out to thoroughly investigate the effects of peer discouragement, given that limited data exist.

### Limitations

This study contains several limitations that are worth mentioning. Firstly, the respondents were recruited from a previous cross-sectional study. This limited our sampling variability. Secondly, COVID-19 has been well-controlled in Hong Kong when compared to other western countries. Thus, the differences in the severity of outbreaks and anti-pandemic measures may result in different lived experiences for young smokers. Therefore, our obtained results may not be able to transfer to a wider population. Thirdly, our findings can only describe the changes and effects of the pandemic on smokers during the study period. It may not be applicable to other stages or periods of the pandemic as it will change over time. Hence, the behaviours of the smokers will be altered accordingly. Future studies are recommended to be conducted at a later period of stage of the pandemic and compare their results with our findings in this study to investigate whether the changes are different, temporary, or permanent. Importantly, it will be of paramount importance to examine if the smokers who quit in this study relapse at a later stage or after the end of the pandemic. Our findings can also provide researchers information about the needs and concerns of young smokers during this period of pandemic. Further studies can target these needs and concerns to design and implement appropriate interventions.

## 5. Conclusions

This qualitative study summarised the lived experience of young smokers in this pandemic, and hence, shed light on our understanding of the reasons underlying mixed patterns of changes in their smoking behaviour during COVID-19. Our findings show that this pandemic and the anti-pandemic measures, i.e., mask mandates, stay-at-home and work-from-home orders, and class suspensions, result in both new obstacles and new advantages for smoking cessation among young people. To inform policy making, more studies should therefore be performed to monitor any transition of tobacco products and the trajectory of use in this population during this pandemic.

## Figures and Tables

**Table 1:** Demographic characteristics and smoking profile of the participants (N = 48).

Age, Mean (SD)	21.4 (2.8)
**Sex**	
Male	34
Female	14
**Education attainment**	
Primary or below	6
Secondary	10
Subdegree	27
Tertiary	5
**Marital status**	
Single	45
Cohabiting/divorced/separated/widowed	1
Missing	2
**Employment status**	
Employed	19
Full-time student	27
Unemployed	2
**Types of tobacco used in the past 30 days**	
Traditional cigarettes	27
Electronic cigarettes and/or heated tobacco products	17
Dual users	11
**Daily cigarette consumption, mean (SD)**	8.2 (6.2)
**Nicotine Dependence by the Fagerstrom test (0–10)**	
Mild (0–3)	28
Moderate (4–5)	13
Severe (6–10)	7
**Age of starting smoking, mean (SD)**	15.8 (2.9)
**Duration of smoking, mean (SD)**	5.7 (3.4)
**Previous quit attempt(s)**	
Yes	29
No	1
**Change in daily cigarette consumption after pandemic**	
Increase	19
Decrease	21
Maintain	8
**Stage of readiness to quit**	
Pre-contemplation	28
Contemplation	13
Preparation	7

SD = Standard Deviation.

## Data Availability

Data will be available upon reasonable request.

## Supplementary Materials

The following is available online at https://www.mdpi.com/article/10.3390/ijerph19074145/s1, Table S1: An abridged table of themes, subthemes, and examples of quotes from the interviews.
