# Supplementary material for: Exploring Factors Contributing to the Smoking Behaviour among Hong Kong Chinese Young Smokers during COVID-19 Pandemic: A Qualitative Study
Source: Int J Environ Res Public Health. 2022 Mar 31;19(7):4145. doi: 10.3390/ijerph19074145 (PMC8998443; doi:10.3390/ijerph19074145)
Supplement: Supplementary file 1 [file ijerph-19-04145-s001.zip › ijerph-1653262-supplementary.pdf]

International Journal of
Environmental Research and Public Health

MDPI

Supplementary Materials

Table S1. An abridged table of themes, subthemes and examples of quotes from the interviews

**Themes**	**Subthemes**	**Examples of quotes**
1. Perceptions of COVID-19 and its association with smoking	1. COVID-19 is serious but does not affect young people too much	'COVID-19 is serious, but not many young people die because of that. Only older people and those with chronic diseases may die after infection. I think the chance (for me) to get COVID is small…' *Respondent M3, male, aged 20*
	2. Relationship between COVID-19 and smoking	'Yes, the government said smoking can increase the chance of getting COVID. However, there are many things which can increase the risk of infection. For example, going out with friends, or waiting for a bus at the bus stop. You can only avoid every risky activity by staying at home. This is impossible. That's why I continue to smoke' *Respondent M5, female, aged 22*
	3. Protect family members from infection	'I personally don't worry about infection. Even if I am infected, the possibility of death is low as I am young. (But why did you decide to quit smoking?) My father has multiple chronic diseases—diabetes and hypertension. I heard from the news that smokers are more likely to be infected with COVID-19. I don't want to be infected and then pass the disease to my family, especially my father. That's why I stopped smoking.' *Respondent D12, male, aged 23*

2. More time at home	1. Easier to access tobacco-related products	'I smoked more in this pandemic… Because I stay at home most of the time. […] Because of class suspension, I don't have to go to school. Previously, even if I wanted to smoke, I couldn't do that because it breaks the school rules. However, when I am at home, I can smoke whenever I want. I can easily reach cigarettes. Hence, I smoke more.' *Respondent I6, female, aged 19*
	2. Boredom	'Because classes have been suspended, I have spent most of the time at home. However, staying at home is boring. Sometimes, I watch YouTube videos to kill time. When watching the videos, I always smoke. […] No particular reason; I just smoke unconsciously when watching videos. Habit. Hence, I smoked more.' *Respondent I12, male, aged 23*
	3. Increased family conflicts	'My relationship with my father is not good. However, the situation was still acceptable as previously he had to go out for work, and we didn't meet each other frequently. […Now it is] Worse. Due to COVID, he stays at home most of the time. I also stay at home because my school has been closed. So my father always picks on me regardless what I do. I feel so stressed and annoyed, so I smoke more.' *Respondent I8, male, aged 20*
	4. Reduced smoking because family members don't know I smoke	'My parents do not know I am a smoker. As I am required to attend online classes via Zoom at home, I cannot go outside to smoke, and hence reduced smoking.' Respondent D4, female, aged 18
3. Taking masks off to smoke	1. This is illegal	'I used to smoke on the street because my parents did not know of my smoking habit. However, that has become difficult since the pandemic. As you know, taking off masks is illegal. The police patrols frequently on the weekends. If they find you taking off masks for

		smoking, you have to pay $5,000 [as a fine]. This is a lot for me. Hence I have reduced smoking.' Respondent D9, male, aged 21
	2. Inconvenience	'Now, all people in Hong Kong are required to wear masks [in public areas]. If I smoke, I have to take off my mask, and hold the mask throughout smoking. On one occasion, I dropped my mask on the street when I smoked. And I forgot to bring another mask with me. Luckily, a smoker gave a new one (mask) to me. It is so inconvenient.' Respondent D15, female, aged 24
	3. Social stigma	'Almost 100% of people in Hong Kong wear a mask. People will stare you if you do not wear mask regardless of any reason. If you take off your mask on the street, the surrounding people will stay far away and walk very fast. This feeling is not good.' Respondent D18, male, aged 25
4. COVID-19 has severely affected smokers' daily lives	1. Quit or reduced smoking to save money	'My job is not secure. […] I am working in the retail industry. My company has been greatly affected after the border was closed. Some of my colleagues were made redundant. I don't know how long my company can survive. So, I want to save more money in case I am made redundant. Cigarettes are expensive. The only way is not to buy so I can save money. I won't die if I don't smoke.' Respondent D21, male, aged 25
	2. Smoking offers respite from financial distress	'There's nothing you can do. All people in the world face the same situation. [Did you talk to your friends?] Meaningless. Things would not be improved. [So you smoked?] Yes. This is the only thing that I can do. It provides me a space to breathe. I can stop thinking about

		this issue (redundancy) for a while. [So this makes you smoke more?] Yes.' Respondent I14, female, aged 23
	3. Worry about their performance in public examinations	'I hope everything can return to normal as soon as possible because I have to attend the DSE [Hong Kong Diploma of Secondary Education, a public exam] next year. But now, we can only go to school for half-days. This is not enough for us to prepare for the DSE. I am so worried that I cannot do well in the DSE and then will not get an offer from the university. This makes me stressed, and I smoke more.' Respondent I1, male, aged 17
5. Social gatherings	1. Reduced smoking due to less peer pressure	'Before COVID, I was always smoking with my peers because they asked me to in the gatherings. They asked me to smoke, and so I smoked. […] I thought of [not smoking] before. But I was so scared that they would not invite me to play the next time. I do not want to be alone. [Why would you reduce your smoking consumption during COVID-19?] Actually, I did not think of reducing smoking. However, during COVID, my parents asked me not to go out so often. Also, the school has been closed. So I have fewer gatherings with them [smoking peers]. That's why I smoked less.' Respondent D6, male, aged 19
	2. Increased smoking due to less social support	'After COVID, I met them [my friends] less often. Maybe one time a month. I did not intend to smoke more. But there was no one to remind me [not to smoke], hence I smoked whenever I wanted. So it seems that I smoke more now.' Respondent I13, female, aged 23
6. Access to tobacco products	1. Inconvenient to buy electronic cigarettes and heated tobacco products	'It is difficult to buy EC in the pandemic. They are only available in checkered shops in Mong Kok. However, the shops now close at 8:00 pm due to less foot traffic. Because I get

off work at 6:00 pm, it would be a rush for me to buy EC. So I switched to smoking conventional cigarettes.' Respondent I14, female, aged 23
